# Serum creatinine levels, traditional cardiovascular risk factors and 10-year cardiovascular risk in Chinese patients with hypertension

**DOI:** 10.3389/fendo.2023.1140093

**Published:** 2023-03-16

**Authors:** Xin Chen, Hang Jin, Dan Wang, Jiali Liu, Yu Qin, Yongqing Zhang, Yuqing Zhang, Quanyong Xiang

**Affiliations:** ^1^ School of Public Health, Southeast University, Nanjing, Jiangsu, China; ^2^ Department of Chronic Non-Communicable Disease Control, Jiangsu Province Center for Disease Control and Prevention, Nanjing, Jiangsu, China; ^3^ Department of Cardiology, Nanjing Jiangning Hospital, The Affiliated Jiangning Hospital of Nanjing Medical University, Nanjing, Jiangsu, China

**Keywords:** serum creatinine, cardiovascular disease, risk factor, China-PAR model, hypertension patients

## Abstract

**Background:**

Serum creatinine is associated with cardiovascular risk and cardiovascular events, however, the relationship between serum creatinine levels and cardiovascular risk is not well established in hypertensive population in Jiangsu Province. We aimed to evaluate the association of serum creatinine levels with traditional cardiovascular risk factors and 10-year cardiovascular risk in a Chinese hypertensive population.

**Methods:**

Participants were patients with hypertension registered and enrolled in health service centers in 5 counties or districts from January 2019 to May 2020 in Jiangsu Province of China followed strict inclusion and exclusion criteria, demographics as well as clinical indicators and disease history and lifestyle were collected. Participants were divided into four groups according to quartiles of serum creatinine levels, then the China-PAR model was used to calculate 10-year cardiovascular risk for each individual.

**Results:**

A total of 9978 participants were enrolled in this study, 4173(41.82%) were males. The blood pressure level and prevalence of dyslipidemia, elderly, current smokers and drinking as well as obesity were higher in the Q4 group than the Q1 group (all *P* < 0.05). Multivariable logistic regression showed that serum creatinine in the Q4 group compared with that in the Q1 group was positively associated with overweight and obesity (OR=1.432, 95% CI 1.237-1.658, *P*<0.001), while negatively associated with physical activity (OR=0.189, 95%CI 0.165-0.217, *P*<0.001), and so on. Multiple linear regression showed 10-year cardiovascular risk is positively associated with serum creatinine levels after adjusting for multiple risk factors (β=0.432, *P*< 0.001).

**Conclusion:**

Serum creatinine was associated with several traditional cardiovascular risk factors and the 10-year cardiovascular risk in hypertensive patients. Creatinine-reduction and kidney-sparing therapy are essential for patients with hypertension to optimize control of cardiovascular risk.

## 1 Introduction

Serum creatinine (Scr) is the anhydride form of creatine and serves a marker of renal function, which mainly comes from muscle metabolism (1). In clinical studies, elevated serum creatinine levels are generally considered as an adverse events or outcomes, often indicating renal impairment (2, 3), meanwhile, studies have shown that impaired renal function is often accompanied by increased cardiovascular risk (4). Serum creatinine levels are not only a contributor to the development cardiovascular events, but also strongly associated with longitudinal risk for cardiovascular disease (CVD) and mortality (5, 6).

Previous studies have shown that slight changes in serum creatinine incrementally associated with increased risk for CVD such as coronary heart disease and heart failure (6, 7), but their association with 10-year cardiovascular risk has not been evaluated in patients with essential hypertension in China. It has been reported that serum creatinine is significantly correlated with pre-inflammatory markers such as Lipoprotein (a)(Lp(a)) and high sensitive C Reactive Protein (hs-CRP) (8, 9), however, the relationship between serum creatinine levels and traditional cardiovascular risk factors such as diabetes and dyslipidemia remains controversial.

The prediction model for atherosclerotic cardiovascular disease (ASCVD) risk in China (China-PAR) has been validated in several Chinese population cohort, which is considered to be a suitable cardiovascular risk prediction model for Asians (10–12). Based on the risk score calculated by the China-PAR risk prediction model, participants were classified into different risk levels and then subsequently treated with corresponding intervention measures. Because of the interaction between serum creatinine and cardiovascular events, we suspected that serum creatinine might be associated with the predicted 10-year cardiovascular risk. However, the data on the association between serum creatinine and predicted 10-year cardiovascular risk is fairly limited.

Therefore, the aim of our study is to estimate the association between serum creatinine levels and traditional cardiovascular risk factors and 10-year cardiovascular risk based on data from a hypertensive population in Jiangsu Province of China, in order to provide references for the prevention of CVD in hypertensive patients with higher creatinine levels.

## 2 Method

### 2.1 Data source and participants

**Figure 1:**
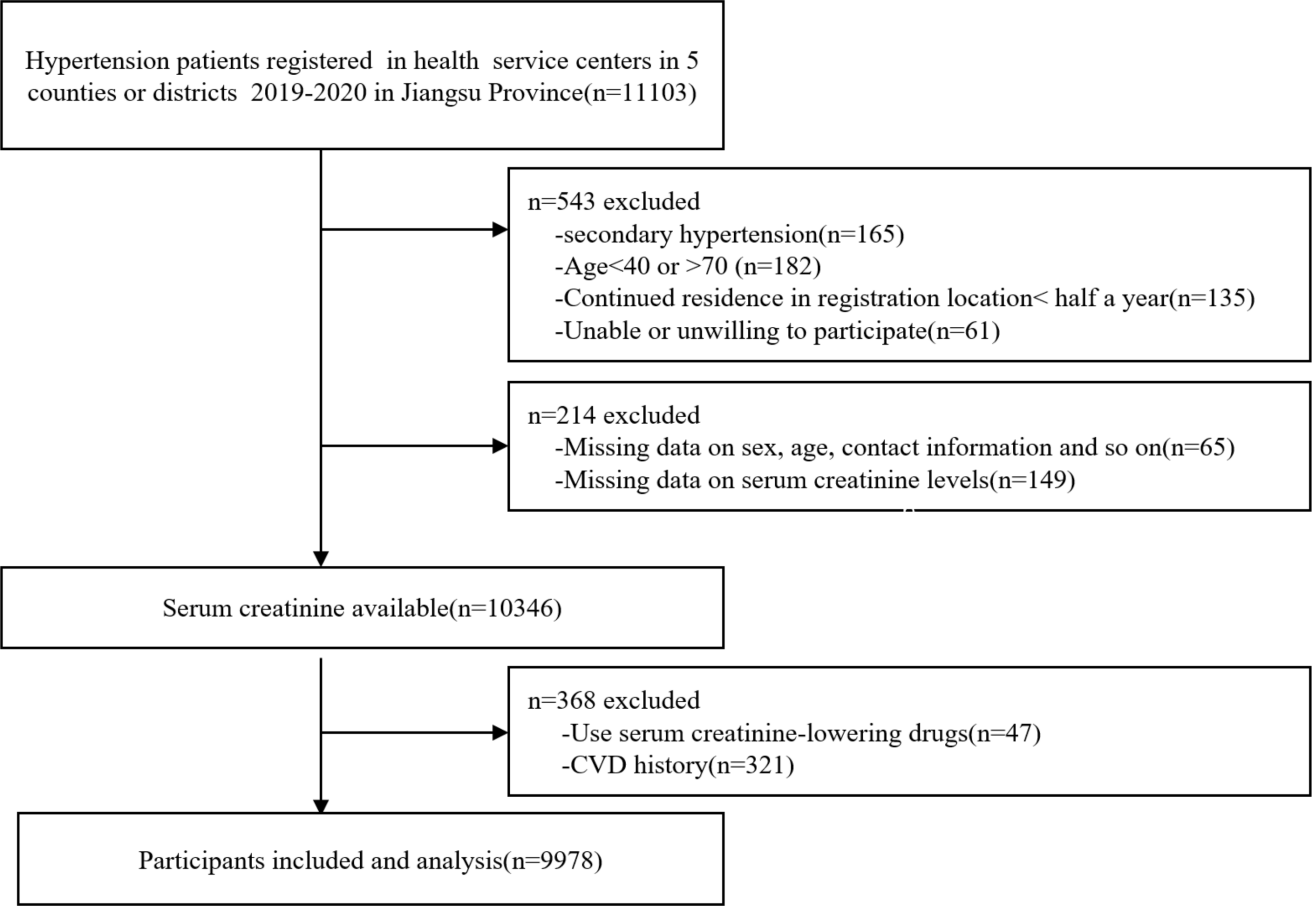
Flow chart of participants. CVD, cardiovascular disease.

Five representative counties or districts in Jiangsu province were selected by multistage stratified random sampling method according to the characteristics of regional economic development (north, midland and south Jiangsu Province), population distribution and lifestyle to ensure the representativeness of research participants. Participants with essential hypertension were registered and enrolled from 50 towns or communities (10 towns or communities were randomly selected according the Random number table method from each counties or districts) in health service centers from January 2019 to May 2020 in Jiangsu Province of China followed strict inclusion and exclusion criteria. Participants aged 40-70 years old, continued residence in registration location for more than half a year, and voluntarily signed informed consent were included. The exclusion criteria were lacking of basic information, diagnosed with secondary hypertension or coronary heart disease, stroke and heart failure and other CVD, used serum creatinine-lowering agents or other drugs that may affect creatinine levels and inability or unwillingness to participate in the survey. A total of 9978 participants with essential hypertension were included in the final analysis (**Figure 1**). Traditional cardiovascular risk factors such as height and weight, blood pressure and waist circumference (WC), as well as disease history such as diabetes and dyslipidemia, and lifestyle factors such as smoking, drinking, levels of vegetables and fruits intake were collected. Participants were divided into four groups according to quartiles of serum creatinine levels: quartile1 (Q1) group (Scr ≤ 68.00 µmol/l, n=2414), quartile 2 (Q2) group (Scr 68.01-78.00 µmol/l, n=2470), quartile 3 (Q3) group (Scr 78.01-89.99µmol/l, n=2540), and quartile 4 (Q4) group (Scr ≥ 90.00µmol/l, n = 2554). The procedures followed in this study were approved by the Ethics Review Board of Jiangsu Center for Disease Control and Prevention (SL2015-B004-01). Informed consent was obtained from all participants, and all study procedures were conducted in accordance with the Strengthening the Reporting of Observational Studies in Epidemiology guidelines, and complied with the principles of the Declaration of Helsinki (1975, revised 2013).

### 2.2 Serum creatinine measurement

The participants are required to control diet and not to over eat meat or do strenuous exercise within three days prior to the measurement. Blood samples were obtained from an antecubital vein after fasting for at least 8 hours and then aliquoted within 2 hours and frozen at −80°C, then transported in dry ice to the central laboratory in Jiangsu Province Center for Disease Control and Prevention, which was certificated by The National Laboratory Certification of China. The serum creatinine was measured by automatic biochemical analyzer (Abbott Laboratories, USA) within one week, and the whole process of laboratory testing was strictly controlled by professionals.

### 2.3 Definition of traditional cardiovascular risk factors

In our study, hypertension was defined as systolic blood pressure (SBP) ≥140 mmHg and/or diastolic blood pressure (DBP) ≥90 mmHg or with anti-hypertensive treatment or participants had a reported history of hypertension (13). Hypertension treatment was defined as regularly taking antihypertensive drugs within two weeks before investigation. Diabetes was defined as fasting plasma glucose (FPG)≥7.0 mmol/L or participants are taking hypoglycemic drugs or have a history of diabetes (14). Dyslipidemia was defined as total cholesterol (TC)≥6.22 mmol/L and/or triglyceride (TG)≥2.26 mmol/L and/or high density lipoprotein-cholesterol (HDL-C) <1.04 mmol/L and/or low density lipoprotein-cholesterol (LDL-C) ≥4.14 mmol/L, or participants reported a history of dyslipidemia or were taking lipid-lowering drugs (15). Body mass index (BMI) is calculated as weight divided by the square of height and then be divided into four grades: <18.5 kg/m^2^ as the underweight group; 18.5–23.9 as the normal group; 24.0–27.9 as the overweight group; ≥28.0 kg/m^2^ as the obesity group (16, 17). Smoking was defined as participants smoked one or more cigarettes per day during the 30 days prior to the survey (18). Drinking was defined as having consumed alcohol in the 30 days before the survey and at least once a week (19).

### 2.4 Risk estimation model

The China-PAR model was adopted to calculate the 10-year cardiovascular risk for each individual, which is available online (https://www.cvdrisk.com.cn/ASCVD/ Articles/Index/52). The China-PAR risk score included sex, age, SBP, WC, smoking, diabetes, geographic region, urbanization, family history of CVD, and treatment for hypertension. Based on the calculated risk score, the participants were divided into three risk categories: <5% as low risk group, 5-9.9% as moderate risk group, and ≥10% as high risk group (10).

### 2.5 Statistical analysis

Continuous variables meeting the normal distribution were expressed using the mean ± standard deviation (SD) and use F-test. Categorical variables are expressed as counts and percentages and were compared using *x^2^-*test. Multivariate logistic regression analysis was used to determine the association of serum creatinine with cardiovascular risk factors. Sensitivity analysis excluded the participants receiving hypertension treatment and diabetes, and multiple linear regression analysis was used to study the relationship between serum creatinine level and 10-year cardiovascular risk. Statistical analyses were performed using SPSS version 27.0(IBM, Armonk, NY, United States), and two-sided *P* values of <0.05 were considered statistically significant.

## 3 Results

### 3.1 Characteristics of participants

The general characteristics of the 9978 participants enrolled in this study by serum creatinine levels are presented in **Table 1**. Among all participants, the average age was 58.58 years, with male gender distribution of 41.82%. Results of the study grouped by serum creatinine quartiles were indicated that the blood pressure level and prevalence of dyslipidemia, elderly, current smokers and drinking as well as obesity were higher in the Q4 group than the Q1 group (all *P*<0.05).

**Table 1:** Characteristics of participants according to quartiles of serum creatinine.

Variables	All the participants (n=9978)	Quartiles of Serum creatinine (Scr)				*P* value
		Q1 group (Scr ≤ 68.00 µmol/l, n = 2414)	Q2 group (Scr68.01-78.00 µmol/l, n = 2470)	Q3 group (Scr78.01-89.99µmol/l, n = 2540)	Q4 group (Scr≥90.00µmol/l, n = 2554)	
Demographics characteristics						
Age, years	58.58 ± 7.30	57.89 ± 7.30	58.68 ± 7.24	58.46 ± 7.41	59.26 ± 7.17	<0.001
Age, %						
<60	4935(49.46)	1311(54.31)	1223(49.51)	1270(50.00)	1131(44.28)	<0.001
≥60	5043(50.54)	1103(45.45.69)	1247(50.49)	1270(50.00)	1423(55.72)	
Male, %	4173(41.82)	512(21.21)	825(33.40)	1098(43.23)	1738(68.05)	<0.001
Rural, %	8678(86.97)	1984(82.19)	2213(89.60)	2294(90.31)	2187(85.63)	<0.001
Clinical characteristics						
SBP, mm Hg	150.79 ± 11.95	149.45 ± 10.82	150.73 ± 11.25	151.33 ± 12.27	151.57 ± 13.13	<0.001
DBP, mm Hg	94.16 ± 7.37	92.71 ± 6.59	93.80 ± 7.22	94.75 ± 7.36	95.30 ± 7.93	<0.001
WC, cm	89.40 ± 10.14	87.53 ± 10.05	89.08 ± 10.39	90.02 ± 10.13	90.87 ± 9.70	<0.001
BMI, %						
Underweight <18.5 kg/m^2^	107(1.07)	37(1.53)	36(1.46)	21(0.83)	13(0.51)	<0.001
Normal 18.5–23.9 kg/m^2^	2407(24.12)	659(27.30)	654(26.48)	572(22.52)	522(20.44)	
Overweight 24.0–27.9 kg/m^2^	4287(42.96)	1009(41.80)	1042(42.19)	1099(43.27)	1137(44.52)	
Obesity ≥28.0 kg/m^2^	3177(31.84)	709(29.37)	738(29.88)	848(33.39)	882(34.53)	
Heart rate, bpm	73.52 ± 8.41	74.12 ± 6.90	73.37 ± 7.97	73.37 ± 9.21	73.26 ± 9.24	0.001
Diabetes, %	614(6.15)	138(5.72)	150(6.07)	162(6.38)	164(6.42)	0.714
Dyslipidemia, %	3511(35.18)	787(32.60)	821(33.24)	910(35.83)	993(38.88)	<0.001
TC, mmol/L	4.86 ± 1.92	4.66 ± 1.98	4.75 ± 0.93	4.89 ± 1.05	5.10 ± 2.94	<0.001
TG, mmol/L	1.83 ± 1.33	1.80 ± 1.35	1.81 ± 1.32	1.83 ± 1.25	1.88 ± 1.42	0.239
HDL-C, mmol/L	1.64 ± 0.54	1.70 ± 0.54	1.63 ± 0.46	1.63 ± 0.59	1.61 ± 0.54	<0.001
LDL-C, mmol/L	2.70 ± 0.89	2.71 ± 0.92	2.69 ± 0.87	2.69 ± 0.88	2.71 ± 0.89	0.794
Course of hypertension, %						
< 5 years	6040(60.53)	1593 (65.99)	1563(63.28)	1498(59.98)	1386(54.27)	<0.001
≥5 years	3938(39.47)	821(34.01)	907(36.72)	1042(40.02)	1168(45.73)	
Hypertension treatment, %	8156(81.74)	1953(80.90)	1953(78.07)	2063(81.22)	2187(85.63)	<0.001
Lifestyle characteristics						
Smoking, %	1899(19.03)	237(9.73)	395(15.99)	506(19.92)	761(29.80)	<0.001
Drinking, %	1632(16.36)	179(7.42)	314(12.71)	433(17.05)	706(27.64)	<0.001
Intake of vegetables and fruits, %						
≤400g/d	3320(33.27)	769(31.86)	794(32.15)	826(32.52)	931(36.45)	0.001
>400g/d	6658(66.73)	1645(68.14)	1676(67.85)	1714(67.48)	1623(63.55)	
Limiting intake of high fat cholesterol foods, %	5198(52.09)	1243(51.49)	1291(52.27)	1302(51.26)	1362(53.33)	0.449
Physical exercise, %						
<3 times/week	3950(39.59)	473(20.42)	954(39.62)	1198(47.17)	1325(51.88)	<0.001
≥3 times/week	6028(60.41)	1941(80.41)	1516(61.38)	1342(52.83)	1229(48.12)	

Normal continuous variables were expressed using the mean ± SD. Categorical variables were reported as the counts and percentages. SBP, systolic blood pressure; DBP, diastolic blood pressure; WC, waist circumference; BMI, body mass index; TC, total cholesterol; TG, triglyceride; HDL-C, high density lipoprotein-cholesterol; LDL-C, low density lipoprotein-cholesterol.

### 3.2 Association between serum creatinine levels and cardiovascular risk factors

Multivariable logistic regression was performed to study the relationship between serum creatinine and cardiovascular risk factors, as shown in **Table 2**, in total participants, the odds ratio (OR) for age, overweight and obesity, drinking, physical exercise, and intake of vegetables and fruits and so on, were 1.403(95% CI 1.237-1.592, *P*<0.001), 1.432(95%CI 1.237-1.658, *P*<0.001), 1.336(95% CI 1.080-1.653, *P*=0.008), 0.189(95%CI 0.165-0.217, *P*<0.001) and 0.727(95%CI 0.637-0.829, *P*<0.001) in the Q4 group compared with that in the Q1 group respectively, which suggests that serum creatinine levels are positively correlated with age, obesity and alcohol consumption, but negatively correlated with fruits and vegetables intake.

**Table 2:** Association between serum creatinine and cardiovascular risk factors.

Variables		OR (95%CI)	*P* value
Age	<60	1(Reference)	
	≥60	1.403(1.237,1.592)	<0.001
Male		9.775(8.316,11.490)	<0.001
Rural		1.517(1.265,1.819)	<0.001
SBP		0.997(0.991,1.003)	0.280
DBP		1.035(1.025,1.046)	<0.001
BMI	<24	1(Reference)	
	≥24	1.432(1.237,1.658)	<0.001
Heart rate		0.991(0.983,0.998)	0.013
Dyslipidemia		1.311(1.150,1.494)	<0.001
Course of hypertension≥5 years		1.530(1.341,1.746)	<0.001
Hypertension treatment		1.320(1.115,1.564)	0.001
Smoking		1.053(0.863,1.284)	0.610
Drinking		1.336(1.080,1.653)	0.008
Intake of vegetables and fruits>400g/d		0.727(0.637,0.829)	<0.001
Physical exercise≥3 times/week		0.189(0.165,0.217)	<0.001

OR, odds ratio; CI, confidence interval; SBP, systolic blood pressure; DBP, diastolic blood pressure; WC, waist circumference; TC, total cholesterol; HDL-C, high density lipoprotein-cholesterol.

### 3.3 Association between serum creatinine and cardiovascular risk factors according gender stratification

**Figure 2:**
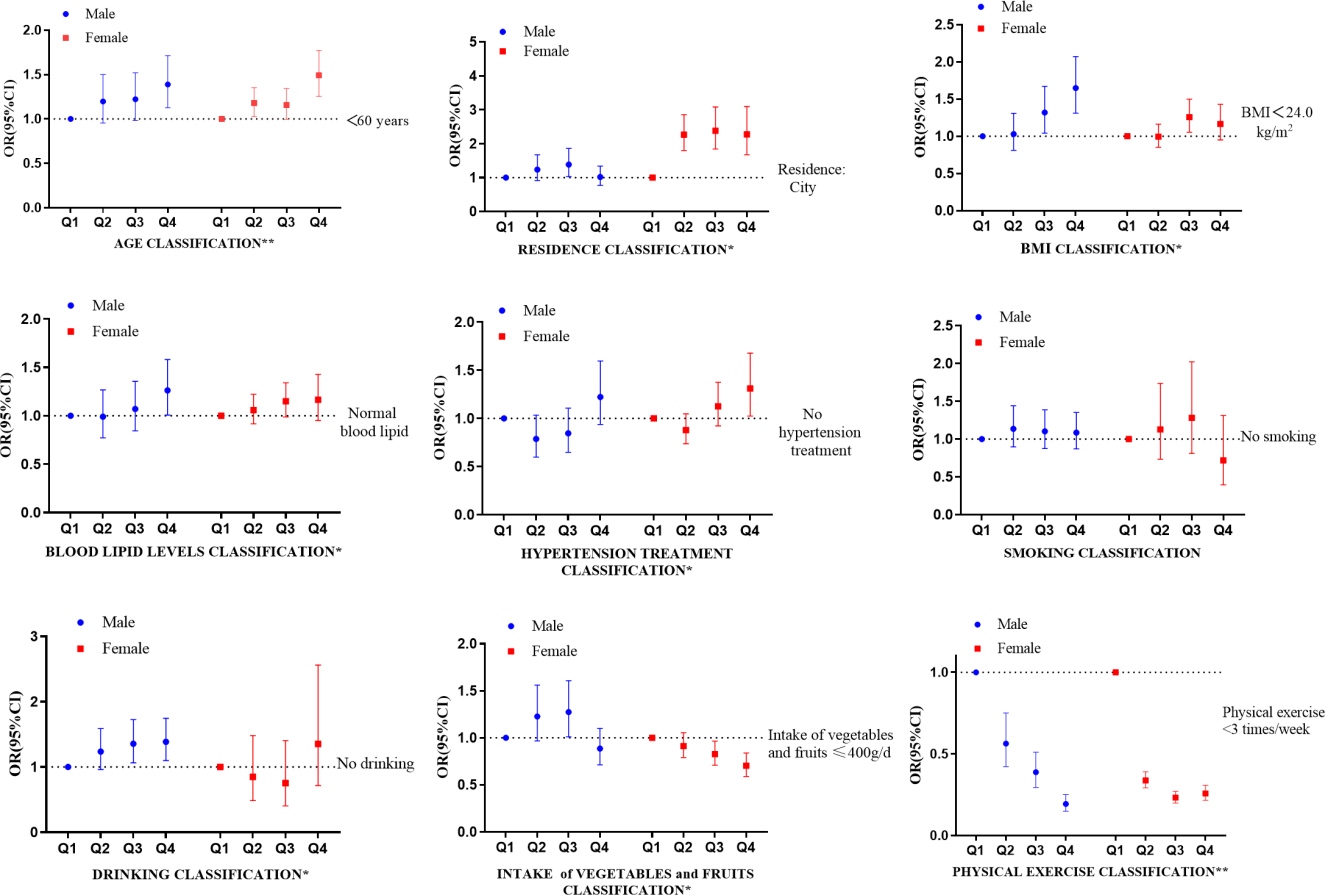
Association between serum creatinine and cardiovascular risk factors according to gender stratification. CI, confidence interval. *The difference in males or females was statistically significant (the Q4 group vs. the Q1 group, *P* < 0.05). **The difference both in males and females were statistically significant (the Q4 group vs. the Q1 group, *P*<0.05).

Results further analyzed according to gender stratification was shown in **Figure 2**, the OR for age, drinking and physical exercise was 1.389 (95% CI 1.126-1.714, *P* < 0.05), 1.385(95% CI 1.098-1.747, *P*< 0.05), 0.194(95% CI 0.149-0.251, *P*<0.05) in the Q4 group compared with that in the Q1 group in men, respectively.

As for women, the OR for age, hypertension treatment, physical exercise and intake of vegetables and fruits was 1.492(95% CI 1.255-1.773, *P* < 0.05), 1.310(95% CI 1.024-1.677, *P*<0.05), 0.258(95%CI 0.216-0.308, *P*<0.05), 0.704(95%CI 0.590-0.839, *P*<0.05) in the Q4 group compared with that in the Q1 group, respectively. The results showed that serum creatinine was positively associated with age, while negatively associated with physical activity in both genders.

### 3.4 10-year cardiovascular risk according to quartiles of serum creatinine

**Figure 3:**
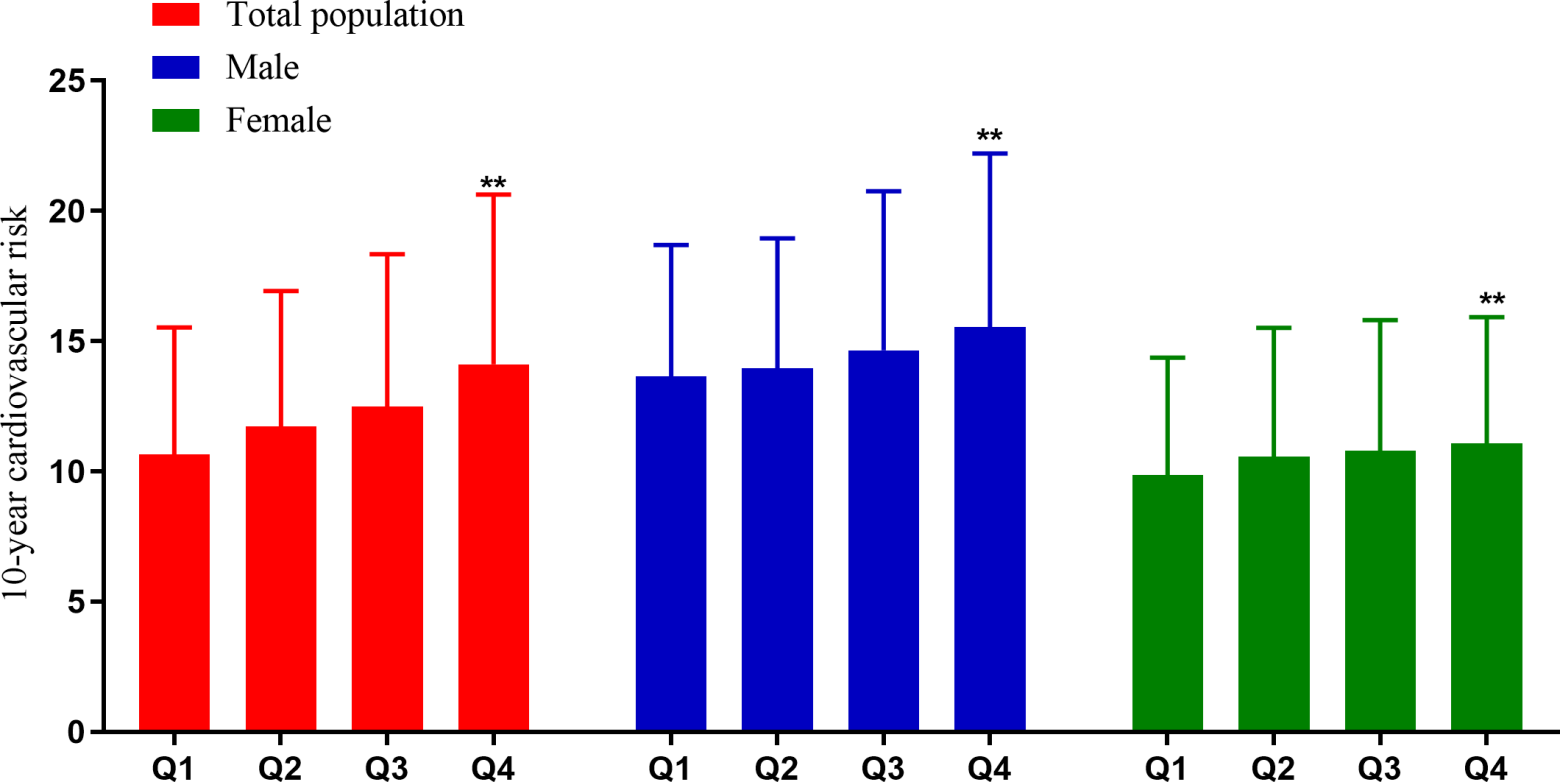
Cardiovascular risk according to quartiles of serum creatinine. **P < 0.001 (the Q4 group vs. the Q1 group).

The 10-year predicted cardiovascular risk according to quartiles of serum creatinine is shown in **Figure 3**. In the total participants, the average risk of CVD in Q1 and Q4 groups were 10.57% and 14.10%, respectively, and the difference was statistically significant (*P* < 0.001). The mean risk of CVD in Q1 and Q4 groups were 13.56% and 15.53% in men, and 9.76% and 11.07% in women, respectively (all *P* < 0.001).

### 3.5 Distribution of 10-year cardiovascular risk classes according to quartiles of serum creatinine

**Figure 4:**
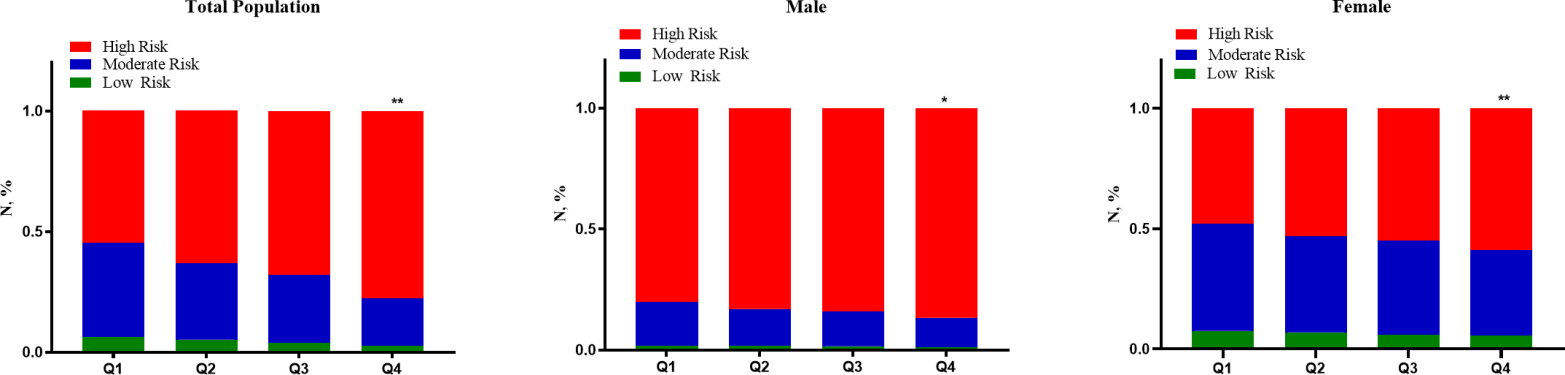
Distribution of cardiovascular risk classes according to quartiles of serum creatinine. *P < 0.05 (the Q4 group vs. the Q1 group). **P < 0.001 (the Q4 group vs. the Q1 group).

The distribution of cardiovascular risk classes according to quartiles of serum creatinine is shown in **Figure 4**. In the total participants, the proportions of participants with high predicted risk in Q1 and Q4 groups were 54.18% and 77.56%, respectively, and the difference was statistically significant (*P* < 0.001). After grouping by gender, the proportions of individuals at high risk in Q1 and Q4 groups were 80.07% and 86.42% in males, and 47.21% and 58.70% in females, respectively (all *P* < 0.05).

### 3.6 Association between serum creatinine level and 10-year cardiovascular risk

The results of the multiple linear regression are shown in **Table 3**. In the total participants, 10-year cardiovascular risk derived from the China-PAR model is positively associated with serum creatinine levels after adjusting for multiple risk factors in Model 3 (β = 0.432, *P*<0.001). Comparing by gender, cardiovascular risk was positively correlated with serum creatinine level in men (β = 0.504, *P*=0.001), although no statistical significance has been found in women.

**Table 3:** Association between serum creatinine level and cardiovascular risk.

	Model 1		Model 2		Model 3	
	Beta coefficients	*P* value	Beta coefficients	*P* value	Beta coefficients	*P* value
Total participants	2.502	<0.001	0.920	<0.001	0.432	<0.001
Male	1.337	<0.001	1.268	<0.001	0.504	0.001
Female	0.733	<0.001	0.409	<0.001	0.165	0.131

Model 1: uncorrected covariates; Model 2 was adjusted for age and sex; Model 3 was adjusted for Model 2 plus heart rate, dyslipidemia, smoking, drinking, intake of vegetables and fruits, physical exercise and residence, diabetes and hypertension treatment.

### 3.7 Sensitivity analysis

In order to exclude the confounding effects of hypertension treatment and diabetes on serum creatinine, we conducted sensitivity analysis and excluded hypertension patients with hypertension treatment or diabetes, as shown in **Table 4**. After adjusting various factors, the serum creatinine level was positively correlated with 10-year cardiovascular risk in participants without receive antihypertensive drugs (β=1.337, *P*< 0.001), especially in men (β=0.602, *P*< 0.001). In addition, serum creatinine is positively correlated with cardiovascular risk in hypertensive patients without diabetes (β=0.490, *P*< 0.001) in Model 3, comparing by gender, serum creatinine and 10-year cardiovascular risk are positively correlated in both men (β=0.569, *P*< 0.001) and women (β=0.209, *P*=0.046), respectively.

**Table 4:** Association between serum creatinine level and cardiovascular risk (sensitivity analysis).

Sensitivity analysis	Model 1		Model 2		Model 3	
	Beta coefficients	*P* value	Beta coefficients	*P* value	Beta coefficients	*P* value
Participants without hypertension treatment	2.522	<0.001	0.810	<0.001	1.337	<0.001
Male	1.243	<0.001	1.194	<0.001	0.602	<0.001
Female	0.560	0.004	0.269	0.084	0.114	0.352
Participants without diabetes	2.574	<0.001	0.933	<0.001	0.490	<0.001
Male	1.360	<0.001	0.346	<0.001	0.569	<0.001
Female	0.696	<0.001	0.421	<0.001	0.209	0.046

Model 1: uncorrected covariates; Model 2 was adjusted for age and sex; Model 3 was adjusted for Model 2 plus heart rate, dyslipidemia, smoking, drinking, intake of vegetables and fruits, physical exercise and residence, diabetes and hypertension treatment.

## 4 Discussion

The morbidity and mortality of CVD have been increasing gradually in China, partly attributed to the increased exposure and aggregation of multiple cardiovascular risk factors (20). Previous studies have shown that prevention and control measures based on risk factors can effectively reduce cardiovascular risk (21). Elevated creatinine levels have been reported to play a role in the increased risk of a variety of CVD, in addition, some clinical studies have found that increased serum creatinine levels, which often indicate a decrease in glomerular filtration rate, may be used as a predictive marker for CVD (22, 23), similarly, our study showed that the average risk in serum creatinine levels Q1 and Q4 groups were 10.57% and 14.10%, and the proportions of participants with high predicted risk of CVD were 54.18% and 77.56% in the total population. Hypertension is an important risk factor in the development of CVD. The relationship between creatinine and multiple cardiovascular risk factors and cardiovascular risk in patients with hypertension needs to be fully elucidated. In our study, serum creatinine levels were found to be strongly associated with cardiovascular risk factors in hypertensive patients.

The results have important clinical significance. Firstly, we used a relatively novel risk prediction model to evaluate individual cardiovascular risk and provided reference for the control of hypertensive patients with high creatinine level to reduce cardiovascular risk. Secondly, serum creatinine is a relatively convenient biochemical index, and serum creatinine in addition to traditional CVD risk factors should be taken into account when evaluating risk for development of CVD in hypertensive patients, especially in male.

Based on the China-PAR project, the China-PAR risk score has good internal consistency and has been proved to be a suitable method for predicting 10-year cardiovascular risk in Chinese population (10). The 10-year cardiovascular risk for each individual was calculated using the China-PAR risk calculation equation. Hypertension patients are at high risk of CVD, at the same time, the pathogenesis of hypertension is closely related to the kidney, which is not only an important organ for blood pressure regulation, but also one of the target organs of hypertension, studies have shown that hypertensive patients with renal impairment have an increased risk of overall CVD, and even mild renal dysfunction can lead to increased mortality and morbidity of CVD (24). In our study, it was also shown that hypertensive individuals with higher creatinine levels had a higher cardiovascular risk.

The rationale for the positive association between serum creatinine and cardiovascular risk remains to be fully clarified. Serum creatinine refers to endogenous serum creatinine, which is the product of human muscle metabolism and the surrogate of renal function (23). Its level is relatively constant in normal people, when the majority of the human kidney is suffering from pathological damage and the proportion of glomerular filtration rate is decreased (more than 50%), the situation of increased serum creatinine may be clinically apparent, and its concentration depends on many factors such as creatinine production rate, distribution volume, extrarenal metabolism and renal injury (23, 25, 26). Serum creatinine behaves as a marker of pro-inflammatory state, and inflammation-mediated endothelial dysfunction has been shown to be associated with the occurrence of cardiovascular events in women with reduced renal function, in addition, high serum creatinine is often accompanied by a decrease in glomerular filtration rate, which is prone to water-sodium retention and increases the burden on the heart as well as cardiovascular risk (9, 27).

Although this study highlights the association between serum creatinine levels and cardiovascular risk factors and 10-year risk of CVD in patients with hypertension in Jiangsu province of China, there are several limitations. The participants with hypertension were recruited from a single province in China, so extrapolation to other populations should be cautious. In addition, our study is a cross-sectional study, and prospective studies are needed for further verification. Finally, other factors that may affect creatinine levels and cardiovascular risk factors such as urea nitrogen and uric acid were missing in this study, and the number and type of antihypertensive drugs were not considered in the study, but we conducted a sensitivity analysis to reduce part of the confounding effect.

## 5 Conclusions

In conclusion, serum creatinine was associated with several cardiovascular risk factors in a hypertensive population in Jiangsu province. The 10-year cardiovascular risk was higher in hypertensive patients with higher serum creatinine levels, especially in men. Creatinine-reduction and kidney-sparing therapy is essential for patients with hypertension to optimize control of cardiovascular risk factors and reduce cardiovascular risk.

## Data availability statement

The original contributions presented in the study are included in the article/supplementary material, further inquiries can be directed to the corresponding author/s.

## Ethics statement

The studies involving human participants were reviewed and approved by the Ethics Review Board of Jiangsu Center for Disease Control and Prevention (SL2015-B004-01). The patients/participants provided their written informed consent to participate in this study.

## Author contributions

Study concept and design: QX, YuZ, YoZ, YQ, XC; Acquisition of data: XC, DW, HJ, JL, YQ, QX; Analysis and interpretation of data: XC, DW, HJ, JL, YQ, YoZ, QX; Drafting of the manuscript: XC, DW, JL, YQ, QX; Critical revision of the manuscript for important intellectual content: QX, YQ, YuZ; Statistical analysis: XC, DW, JL, YQ, QX; Obtained funding: QX; Technical, or material support: YQ, YoZ, YuZ, QX; Study supervision: QX. All authors contributed to the article and approved the submitted version. 
